# Supplementary material for: Prevalence of iron deficiency anemia in Brazilian women of childbearing age: a systematic review with meta-analysis
Source: PeerJ. 2022 Feb 17;10:e12959. doi: 10.7717/peerj.12959 (PMC8858579; doi:10.7717/peerj.12959)
Supplement: Supplemental Information 2 — MW: Midwest; N: North; NE: Northeast; NOS: Newcastle-Ottawa Scale; S: South; SE: Southeast. [file peerj-10-12959-s002.docx]

**Supplementary Table 1.** Main characteristics of the included studies (n = 91)

| **Reference** | **Year collection** | **Age group** | **Pregnancy** | **Region** | **Sample size** | **Context** | **Measurement method** | **Prevalence (%)** | **NOS summary** |
| --- | --- | --- | --- | --- | --- | --- | --- | --- | --- |
| Américo & Ferraz, 2011 | 2005 to 2008 | Teenagers and adults | Yes | S | 2054 | Basic health unit | Medical record | 6.18 | 2 |
| Araf et al., 2010 | 2001 | Teenagers | No | SE | 105 | School | Cell Dyn 3700 or 4000 hematometer | 3.81 | 4 |
| Araújo, 2012 | 2006 | Teenagers and adults | No | N, NE, MW, S, SE | N: 301; NE: 280;  MW: 299;  S: 283;  SE: 313 | Household survey | Cyanomethemoglobin | N: 14.6; NE: 37.1; MW: 23.1; S: 19.8; SE: 23.3 | 5 |
| Araújo et al., 2013 | 2004 and 2006 | Teenagers and adults | Yes | S | 2004: 366;  2006: 417 | Basic health unit | Cell Dyn 3000 | 2004: 12.3;  2006: 9.3 | 4 |
| Arruda, 1990 | 1989 | Teenagers and adults | Yes | NE | 710 | Basic health unit | Cell Counter CELLM | 30.3 | 2 |
| Arruda, 1997 | 1992 | Not reported | Not reported | NE | 1007 | Not reported | Cyanomethemoglobin | 30.9 | 1 |
| Bagni; Luiz; da Veiga, 2013 | 2008 to 2009 | Teenagers | No | SE | 419 | School | HemoCue | 30.8 | 5 |
| Batista Filho & Romani 1996 | 1996 | Teenagers and adults | No | NE | 1196 | Not reported | Not reported | 24.5 | 1 |
| Bezerra et al., 2018 | 2012 to 2013 | Teenagers and adults | No | NE | 322 | Household survey | Cyanomethemoglobin | 18.6 | 5 |
| Borges et al., 2016 | 2008 | Teenagers and adults | Yes/No | N, NE, MW, S/SE | Not reported | Household survey* | HemoCue Hb 201+ | N: 46.3; NE: 22.8;  MW: 34.8; S/SE: 30.8 | 5 |
| Bresan et al., 2018 | 2017 | Teenagers and adults | Yes | MW | 28 | Indigenous village | Not reported | 23.8 | 2 |
| Bresani et al., 2007 | 2000 to 2001 | Not reported | Yes | NE | 318 | Outpatient | Coulter T 890 | 56.6 | 3 |
| Carvalho et al., 2017 | 2010 to 2015 | Not reported | Yes | NE | 47 | Hospital | Medical record | 14.9 | 1 |
| Cavalcanti et al., 2014 | 2007 | Teenagers and adults | Yes | NE | 5 | Household survey | HemoCue | 60.0 | 3 |
| Cavalcanti et al., 2019 | 2007 to 2008 | Adults | No | NE | 1176 | Outpatient | Sysmex XT-1800i | 12.0 | 6 |
| Cintra, 2018 | 2014 | Teenagers and adults | No | SE | 230 | Household survey | Agabe | 9,6 | 5 |
| Clemente, 2019 | 2006 and 2015 to 2016 | Teenagers and adults | No | NE | 2006: 1480;  2015-2016: 655 | Household survey | HemoCue | 2006: 16.4;  2015-2016: 24.6 | 6 |
| Coelho, 2011 | 1993 to 2007 | Teenagers and adults | Yes | SE | 613 | Hospital | Medical record | 47,8 | 2 |
| Cortês, 2006 | 2004 and 2005 | Teenagers and adults | Yes | MW | 2004: 228;  2005: 228 | Outpatient | HemoCue | 2004: 28.9;  2005: 7.9 | 5 |
| Da Costa et al., 2013 | 2005 | Teenagers | No | SE | 77 | Swim club | Cyanomethemoglobin | 7.8 | 5 |
| Da Silva, 2015 | 2012 | Teenagers and adults | Yes | NE | 349 | Basic health unit | Not reported | 22.6 | 2 |
| Dal Pizzol; Giugliani; Mengue, 2009 | 1991 to 1995 | Adults | Yes | N, NE, S, SE | N:407; NE: 1702; S: 837;  SE: 919 | Outpatient | Medical record | N: 27.0; NE: 35.7; S: 27.1; SE: 28.6 | 4 |
| Dani et al., 2008 | Not reported | Not reported | Yes | S | 102 | Outpatient | Not reported | 13.7 | 3 |
| De Camargo et al., 2013 | 2008 to 2009 | Adults | Yes | MW | 146 | Outpatient | Pentra 80 | 4.8 | 4 |
| De Carli et al., 2018 | 2014 to 2016 | Adults | No | SE | 127 | University | Sysmex XT-2000i | 3.1 | 5 |
| De Castro et al., 2019 | 2017 to 2018 | Adults | No | SE | 27 | Outpatient | Not reported | 37.0 | 1 |
| De França, 2006 | 2005 to 2006 | Teenagers | No | N | 24 | Household survey | Cell Dyn 3500 | 50.0 | 3 |
| De Oliveira; De Barros; Ferreira, 2015 | 2014 | Teenagers and adults | Yes | NE | 428 | Basic health unit | HemoCue | 28.3 | 5 |
| De Sá et al., 2015 | Not reported | Adults | Yes | SE | 54 | Hospital | BC 2800 | 53.7 | 3 |
| De Souza, 2011 | 2009 | Teenagers and adults | Yes/No | MW | 119 | Indigenous village | HemoCue Hb 201 + | 54.6 | 4 |
| Dell’Agno, 2009 | 1999 to 2008 | Teenagers and adults | Yes | S | 32 | Hospital | Medical record | 62.5 | 2 |
| Demétrio; Teles-Santos; dos Santos, 2017 | 2014 to 2015 | Teenagers and adults | Yes | NE | 245 | Basic health unit | Cyanomethemoglobin | 21.8 | 5 |
| Dos Santos, 2018 | 2011 | Adults | No | MW | 174 | Indigenous village | HemoCue Hb 301 | 57.3 | 2 |
| Dos Santos et al., 2020 | 2014 to 2015 | Teenagers and aduls | Yes | N | 220 | Hospital | Cyanide-free photometry | 28.20 | 3 |
| Einloft et al., 2010 | 2004 to 2005 | Teenagers and adults | Yes | SE | 246 | Basic health unit | Medical record | 28.9 | 2 |
| Fabian et al., 2007 | 2003 | Adults | No | S | 252 | Household survey | Cyanomethemoglobin | 21.4 | 6 |
| Fávaro, 2011 | 2010 | Teenagers and adults | Yes/No | NE | Not pregnant: 577;  Pregnant: 20 | Indigenous village | HemoCue | Not pregnant: 16.1;  Pregnant: 55.0 | 5 |
| Ferreira et al., 1998 | Not reported | Teenagers | No | NE | 66 | School | Cell Dyn 3000 CS | 27.3 | 3 |
| Ferreira et al., 2007 | 2004 | Teenager and adults | Yes | N | 100 | Household survey | HemoCue | 11.9 | 3 |
| Ferreira et al., 2008 | 2007 | Teenagers and adults | Yes | NE | 150 | Household survey | HemoCue | 50.0 | 5 |
| Ferreira, 2016 | 2011 to 2014 | Teenagers and adults | No | SE | 608 | Hospital | Medical record | 37.5 | 2 |
| Frota, 2013 | 2010 | Teenagers and adults | No | NE | 978 | Household survey | Agabe | 36.0 | 5 |
| Fujimori; Szarfarc; De Oliveira, 1996 | 1987 | Teenagers | No | SE | 262 | Household survey | Cyanomethemoglobin | 17.6 | 5 |
| Fujimori et al., 1999 | 1993 | Teenagers | Yes | SE | 155 | Outpatient | Cyanomethemoglobin | 14.2 | 2 |
| Fujimori et al., 2011 | 2002 and 2005 to 2008 | Teenagers and adults | Yes | N, NE, MW, S, SE | N (2002): 678;  N (2005-2008): 911;  NE (2002): 2137;  NE (2005-2008): 1718;  MW (2002): 414;  MW (2005-2008): 539;  S (2002): 748;  S (2005-2008): 801;  SE (2002): 2085;  SE (2005-2008): 2088 | Outpatient | Medical record | N (2002): 32.2;  N (2005-2008): 24.9;  NE (2002): 37.4;  NE (2005-2008): 28.7;  MW (2002): 22.2;  MW (2005-2008): 27.8;  S (2002): 7.0;  S (2005-2008): 5.7;  SE (2002): 18.3;  SE (2005-2008): 14.8 | 3 |
| Guerra et al., 1990 | Not reported | Teenagers and adults | Yes | SE | 363 | Outpatient | Cyanomethemoglobin | 12.4 | 4 |
| Hirata et al., 2017 | 2010 | Teenagers | No | SE | 381 | Basic health unit | ABX Pentra 120 | 1.6 | 3 |
| Instituto Nacional de Alimentação e Nutrição (Brasil) | 1997 | Adults | No | SE | 1196 | Household survey | HemoCue | 24.5 | 5 |
| Leite, 1998 | 1997 | Teenagers and adults | Yes/No | MW | 34 | Indigenous village | HemoCue AB | 52.9 | 5 |
| Lerner, 1994 | 1990 | Teenagers | No | SE | 294 | School | Cyanomethemoglobin | 5.4 | 5 |
| Lopes et al., 2006 | 2005 | Teenagers and adults | No | NE | 72 | Hospital | Medical record | 65.3 | 2 |
| Lucyk, 2006 | 2005 | Teenagers and adults | Yes | MW | 170 | Outpatient | HemoCue | 19.4 | 2 |
| Machado et al., 2016 | 2006 and 2008 | Teenagers and adults | Yes | SE | 2006: 259;  2008: 287 | Basic health unit | SysmexXE-2100D | 2006:9.7;  2008: 9.4 | 6 |
| Magalhães et al., 2018 | 2010 to 2011 | Teenagers and adults | Yes | NE | 328 | Outpatient | HemoCue | 18.9 | 5 |
| Mariath et al., 2006 | Not reported | Teenagers | No | S | 167 | School | HemoCue | 31.1 | 5 |
| Marin et al., 2015 | 2011 to 2013 | Adults | No | SE | 200 | Outpatient | Medical record | 11.5 | 2 |
| Marion, 2013 | 2011 | Teenagers and adults | Yes | S | 124 | Outpatient | Not reported | 16.1 | 3 |
| Marques et al., 2015 | 2010 | Adults | No | NE | 140 | University | Cyanomethemoglobin | 79.2 | 5 |
| Massucheti, 2007 | 2003 to 2004 | Adults | Yes | S | 360 | Basic health unit | Medical record | 21.4 | 5 |
| Miranda et al., 2018 | 2015 | Teenagers and adults | Yes | S | 3419 | Hospital | Medical record | 35.9 | 1 |
| Neves, 2018 | 2015 to 2016 | Teenagers and adults | Yes | N | 1445 | Basic health unit | HemoCue | 39.4 | 4 |
| Niquini et al., 2012 | 2008 | Teenagers and adults | Yes | SE | 82 | Basic health unit | Medical record | 15.9 | 4 |
| Orellana et al., 2011 | 2005 | Teenagers and adults | Yes/No | N-MW | Not pregnant: 162;  Pregnant: 11 | Indigenous village | HemoCue AB | Not pregnant: 67.3; Pregnant: 81.82 | 4 |
| Orsolin et al., 2020 | 2015 | Adults | Yes | S | 44 | Hospital | Medical record | 25.0 | 2 |
| Papa et al., 2003 | 2001 to 2002 | Teenagers | Yes | SE | 56 | Outpatient | Cell Dyn 3000 | 21.4 | 2 |
| Rondó; Tomkins, 1999 | 1991 to 1992 | Not reported | Yes | SE | 712 | Hospital | Cyanomethemoglobin | 47.0 | 3 |
| Pereira, 1997 | 1996 | Teenagers and adults | Yes | NE | 515 | Hospital | Cell Dyn | 42.2 | 2 |
| Pereira et al., 2019 | 2014 to 2015 | Teenagers and adults | No | SE | 230 | Day care center | Agabe | 9.6 | 5 |
| Pessoa et al., 2015 | 2004 to 2013 | Teenagers | Yes | SE | 628 | Hospital | Medical record | 41.1 | 3 |
| Pincelli et al., 2018 | 2015 to 2016 | Teenagers and adults | Yes | N | 1101 | Basic health unit/Hospital | Not reported | 40.1 | 3 |
| Pinho-Pompeu et al., 2017 | 2005 to 2013 | Teenagers | Yes | SE | 458 | Outpatient | Medical record | 41.27 | 2 |
| Quintans, 2011 | 2011 | Adults | Yes | NE | 130 | Basic health unit | Medical record | 17.7 | 4 |
| Renz, 2018 | 2009 to 2015 | Adults | Yes | S | 231 | Outpatient | ABX Pentra DX 120 | 25.1 | 3 |
| Rezende, 2007 | 2006 | Teenagers and adults | Yes/No | SE | Not pregnant: 228; Pregnant: 14 | Day care center | HemoCue | Not pregnant: 36.4;  Pregnant: 100.0 | 4 |
| Rocha et al., 2005 | 2002 to 2003 | Teenagers and adults | Yes | SE | 168 | Outpatient | HemoCue | 21.4 | 5 |
| Roncada; Szarfarc, 1975 | 1969 to 1970 | Teenagers and adults | Yes | SE | 56 | Household survey | Not reported | 32.1 | 1 |
| Sales et al., 2021 | 2015 | Teenagers | No | SE | 150 | Household survey | Sysmex XE-2100 | 6.7 | 5 |
| Santos, 2006 | 2005 to 2006 | Teenagers and adults | Yes | NE | 326 | Basic health unit | Cyanomethemoglobin | 31.9 | 5 |
| Santos et al., 2009 | 2006 to 2007 | Adults | Yes/No | S | 865 | Basic health unit | HemoCue | 21.2 | 5 |
| Santos et al., 2012 | 2004 | Teenagers | No | SE | 126 | Outpatient | Medical record | 10.3 | 2 |
| Saunders et al., 2016 | 1999 to 2008 | Adults | Yes | SE | 498 | Hospital | Medical record | 26.3 | 2 |
| Sena de Lira, 2009 | 2005 | Teenagers and adults | No | NE | 1124 | Household survey | HemoCue | 25.1 | 2 |
| Silla et al., 2013 | 2006 to 2007 | Teenagers and adults | No | S | 1999 | Household survey | HemoCue | 36.4 | 5 |
| Silva; Santos; Oliveira, 2018 | 2016 to 2017 | Teenagers | No | NE | 212 | School | HemoCue Hb 301 |  | 4 |
| Silva et al., 2020 | 2017 | Teenagers and adults | Yes | NE | 238 | Hospital | Medical record | 21.7 | 2 |
| Sinisterra-Rodriguez; Szarfarc; Benicio, 1991 | Not reported | Not reported | Yes | SE | 684 | Hospital | Not reported | 27.6 | 1 |
| Szarfarc, 1974 | Not reported | Not reported | Yes | SE | 258 | Hospital | Cyanomethemoglobin | 52.3 | 2 |
| Szarfarc; De Siqueira; Martins, 1982 | Not reported | Not reported | Yes | SE | 151 | Outpatient | Not reported | 22.5 | 2 |
| Szarfarc, 1985 | 1977 to 1981 | Not reported | Yes | SE | 4539 | Outpatient | Cyanomethemoglobin | 35.1 | 3 |
| Tapia et al., 2010 | 2007 to 2008 | Teenagers and adults | Yes | SE | 1448 | Basic health unit | Cell Dyn | 8.3 | 3 |
| Walter et al., 2021 | 2000 to 2020 | Adults | Yes | S | 129 | Outpatient | Not reported | 26.4 | 1 |

MW: Midwest; N: North; NE: Northeast; NOS: Newcastle-Ottawa Scale; S: South; SE: Southeast
