# Supplementary material for: Prevalence of iron deficiency anemia in Brazilian women of childbearing age: a systematic review with meta-analysis
Source: PeerJ. 2022 Feb 17;10:e12959. doi: 10.7717/peerj.12959 (PMC8858579; doi:10.7717/peerj.12959)
Supplement: Supplemental Information 3 [file peerj-10-12959-s003.docx]

**Supplementary Table 2.** Quality assessment of included studies based on the Newcastle-Ottawa Scale

| **Study** | **Domain I** | **Domain II** | **Domain III** | **Domain IV** | **Domain V** | **Total** |
| --- | --- | --- | --- | --- | --- | --- |
|  | Representativeness of the chosen sample | Adequate sample size | Evaluation of non-respondents | Diagnostic criteria | Measurement of biochemical markers |  |
| Américo & Ferraz, 2011 | ★ |  |  | ★ |  | 2 |
| Araf et al., 2010 | ★ |  | ★ | ★ | ★ | 4 |
| Araújo, 2012 | ★★ |  | ★ | ★ | ★ | 5 |
| Araújo et al., 2013 | ★★ | ★ |  | ★ | ★ | 5 |
| Arruda, 1990 |  |  |  | ★ | ★ | 2 |
| Arruda, 1997 |  |  |  | ★ |  | 1 |
| Bagni; Luiz; da Veiga, 2013 | ★★ | ★ |  | ★ | ★ | 5 |
| Batista Filho & Romani 1996 |  |  |  | ★ |  | 1 |
| Bezerra et al., 2018 | ★★ | ★ |  | ★ | ★ | 5 |
| Borges et al., 2016 | ★ | ★ | ★ | ★ | ★ | 5 |
| Bresan et al., 2018 | ★ |  |  | ★ |  | 2 |
| Bresani et al., 2007 | ★ |  |  | ★ | ★ | 3 |
| Carvalho et al., 2017 | ★ |  |  |  |  | 1 |
| Cavalcanti et al., 2014 | ★ |  |  | ★ | ★ | 3 |
| Cavalcanti et al., 2019 | ★★ | ★ | ★ | ★ | ★ | 6 |
| Cintra, 2018 | ★★ | ★ |  | ★ | ★ | 5 |
| Clemente, 2019 | ★★ | ★ | ★ | ★ | ★ | 6 |
| Coelho, 2011 | ★ |  | ★ |  |  | 2 |
| Cortês, 2006 | ★ | ★ | ★ | ★ | ★ | 5 |
| Da Costa et al., 2013 | ★ |  | ★ | ★ | ★★ | 5 |
| Da Silva, 2015 |  | ★ |  | ★ |  | 2 |
| Dal Pizzol; Giugliani; Mengue, 2009 | ★ |  | ★ | ★ | ★ | 4 |
| Dani et al., 2008 | ★ |  |  | ★ | ★ | 3 |
| De Camargo et al., 2013 | ★ |  | ★ | ★ | ★ | 4 |
| De Carli et al., 2018 | ★ | ★ | ★ | ★ | ★ | 5 |
| De Castro et al., 2019 |  |  |  | ★ |  | 1 |
| De França, 2006 | ★ |  |  | ★ | ★ | 3 |
| De Oliveira; De Barros; Ferreira, 2015 | ★★ | ★ |  | ★ | ★ | 5 |
| De Sá et al., 2015 | ★ |  |  | ★ | ★ | 3 |
| De Souza, 2011 | ★ |  | ★ | ★ | ★ | 4 |
| Dell’Agno, 2009 | ★ |  | ★ |  |  | 2 |
| Demétrio; Teles-Santos; dos Santos, 2017 | ★★ | ★ |  | ★ | ★ | 5 |
| Dos Santos, 2018 |  |  |  | ★ | ★ | 2 |
| Dos Santos et al., 2020 |  | ★ |  | ★ | ★ | 3 |
| Einloft et al., 2010 | ★ |  |  | ★ |  | 2 |
| Fabian et al., 2007 | ★★ | ★ | ★ | ★ | ★ | 6 |
| Fávaro, 2011 | ★★ |  | ★ | ★ | ★ | 5 |
| Ferreira et al., 1998 | ★ |  |  | ★ | ★ | 3 |
| Ferreira et al., 2007 |  |  | ★ | ★ | ★ | 3 |
| Ferreira et al., 2008 | ★★ | ★ |  | ★ | ★ | 5 |
| Ferreira, 2016 | ★ |  |  | ★ |  | 2 |
| Frota, 2013 | ★★ | ★ |  | ★ | ★ | 5 |
| Fujimori; Szarfarc; De Oliveira, 1996 | ★★ |  | ★ | ★ | ★ | 5 |
| Fujimori et al., 1999 |  |  |  | ★ | ★ | 2 |
| Fujimori et al., 2011 | ★ | ★ |  | ★ |  | 3 |
| Guerra et al., 1990 | ★ |  | ★ | ★ | ★ | 4 |
| Hirata et al., 2017 | ★ |  |  | ★ | ★ | 3 |
| Instituto Nacional de Alimentação e Nutrição (Brasil), 1998 | ★★ | ★ |  | ★ | ★ | 5 |
| Leite, 1998 | ★★ |  | ★ | ★ | ★ | 5 |
| Lerner, 1994 | ★★ |  | ★ | ★ | ★ | 5 |
| Lopes et al., 2006 | ★ |  |  | ★ |  | 2 |
| Lucyk, 2006 |  |  |  | ★ | ★ | 2 |
| Machado et al., 2016 | ★★ | ★ | ★ | ★ | ★ | 6 |
| Magalhães et al., 2018 | ★★ | ★ |  | ★ | ★ | 5 |
| Mariath et al., 2006 | ★★ | ★ |  | ★ | ★ | 5 |
| Marin et al., 2015 | ★ |  |  | ★ |  | 2 |
| Marion, 2013 | ★ |  |  | ★ | ★ | 3 |
| Marques et al., 2015 | ★★ | ★ |  | ★ | ★ | 5 |
| Massucheti, 2007 | ★★ | ★ | ★ | ★ |  | 5 |
| Miranda et al., 2018 |  |  |  | ★ |  | 1 |
| Neves, 2018 | ★ |  | ★ | ★ | ★ | 4 |
| Niquini et al., 2012 | ★ | ★ | ★ | ★ |  | 4 |
| Orellana et al., 2011 | ★ |  | ★ | ★ | ★ | 4 |
| Orsolin et al., 2020 |  | ★ |  | ★ |  | 2 |
| Papa et al., 2003 |  |  |  | ★ | ★ | 2 |
| Rondó; Tomkins, 1999 | ★ |  |  | ★ | ★ | 3 |
| Pereira, 1997 |  |  |  | ★ | ★ | 2 |
| Pereira et al., 2019 | ★★ | ★ |  | ★ | ★ | 5 |
| Pessoa et al., 2015 | ★ |  | ★ | ★ |  | 3 |
| Pincelli et al., 2018 | ★ |  | ★ | ★ |  | 3 |
| Pinho-Pompeu et al., 2017 | ★ |  |  | ★ |  | 2 |
| Quintans, 2011 | ★ | ★ | ★ | ★ |  | 4 |
| Renz, 2018 | ★ |  |  | ★ | ★ | 3 |
| Rezende, 2007 | ★ |  | ★ | ★ | ★ | 4 |
| Rocha et al., 2005 | ★ | ★ | ★ | ★ | ★ | 5 |
| Roncada; Szarfarc, 1975 | ★ |  |  |  |  | 1 |
| Sales et al., 2021 | ★★ | ★ |  | ★ | ★ | 5 |
| Santos, 2006 | ★★ | ★ |  | ★ | ★ | 5 |
| Santos et al., 2009 | ★ | ★ | ★ | ★ | ★ | 5 |
| Santos et al., 2012 | ★ |  |  | ★ |  | 2 |
| Saunders et al., 2016 | ★ |  |  | ★ |  | 2 |
| Sena de Lira, 2009 | ★ |  |  | ★ |  | 2 |
| Silla et al., 2013 | ★ | ★ | ★ | ★ | ★ | 5 |
| Silva; Santos; Oliveira, 2018 | ★ | ★ |  | ★ | ★ | 4 |
| Silva et al., 2020 | ★ |  |  | ★ |  | 2 |
| Sinisterra-Rodriguez; Szarfarc; Benicio, 1991 |  |  |  | ★ |  | 1 |
| Szarfarc, 1974 | ★ |  |  |  | ★ | 2 |
| Szarfarc; De Siqueira; Martins, 1982 | ★ |  |  | ★ |  | 2 |
| Szarfarc, 1985 | ★ |  |  | ★ | ★ | 3 |
| Tapia et al., 2010 | ★ |  |  | ★ | ★ | 3 |
| Walter et al., 2021 |  |  |  | ★ |  | 1 |
