# Supplementary material for: Prevalence of iron deficiency anemia in Brazilian women of childbearing age: a systematic review with meta-analysis
Source: PeerJ. 2022 Feb 17;10:e12959. doi: 10.7717/peerj.12959 (PMC8858579; doi:10.7717/peerj.12959)
Supplement: Supplemental Information 4 [file peerj-10-12959-s004.docx]

**SYSTEMATIC REVIEW AND/OR META-ANALYSIS RATIONALE**

Among the most common micronutrient deficiencies in women is iron deficiency. During their childbearing age, women are at increased risk of iron deficiency anemia (IDA) due to blood loss from menstruation and often have insufficient dietary iron intake to compensate for menstrual losses. In order to tackle IDA, Brazil has several national programs, such as a food fortification program, which enriches wheat and corn flour with iron and folic acid, and was established in 2004 with the aim to decrease IDA. Collecting data on the iron status of Brazilian women of childbearing age is essential for the development of national public policies and for monitoring the effectiveness of existing programs. However, we are not aware of any systematic review with or without meta-analysis to assess the prevalence of ADI in different contexts with Brazilian women of childbearing age. Knowing the prevalence of IDA in women of childbearing age in different contexts enables the application of more effective strategies to combat this public health problem.
